# Results of a Qualitative Study on Disaster Nursing in a Coastal Region of Lower Saxony/Germany

**DOI:** 10.1093/eurpub/ckac130.075

**Published:** 2022-10-25

**Authors:** F Koppelin, D Palm, K Illiger

**Affiliations:** Jade University of Applied Sciences Oldenburg, TGM, Oldenburg, Germany

## Abstract

The increase in heavy rainfall in recent years shows the need to consider disaster preparedness also for persons in need of assistance and care who are cared for at home or in old people's and nursing homes. Evacuation concepts in the event of a heavy rainfall event lasting several days with simultaneous power failure are hardly available for the vulnerable group so far. As part of the LifeGRID project funded by the Federal Ministry of Education and Research, the question is being investigated as to what regional challenges and requirements arise in the event of flooding and a prolonged power blackout in the Wesermarsch district. In addition, the question will be addressed as to how the current care situation of patients referred to electricity presents itself in such a situation. Within the framework of a qualitative design, expert interviews were conducted with care service managers in winter 2022. The four guided interviews were transcribed and their content analysed. In coping with the assumed catastrophic events, the interviewees see problem areas in the organisation, communication, form of care and target group, in addition to the regional characteristics. It became clear that there are not only unanswered questions regarding responsibilities in the event of a crisis, but also that the diversity of forms of care (e.g. private households) poses a particular challenge. The interviewees do not see any viable alternatives to secure communication in the event of a power failure and also see that respiratory patients, for example, have a special need for care (e.g. due to the limited battery life of the respiratory equipment). The results also show that the nursing experts have different views on how they should prepare for such a scenario. These range from passivity to proactivity. A clear need for action becomes visible for cooperation and networking of the relevant actors, promotion of disaster literacy as well as the necessity to adapt the training curricula.

**Key messages:**

- Climate change is exacerbating the demands for concepts that sustainably contribute to increasing disaster literacy. The results of the study underline this.
- Patients receiving outpatient care and residents of nursing homes for the elderly who depend on a continuous power supply have not yet been the focus of disaster management.

